# Host Abundance Predicts Interactive Roles in Neotropical Bat–Bat Fly Interactions

**DOI:** 10.1002/ece3.73640

**Published:** 2026-05-13

**Authors:** Paulo Mejia, Wesley Dáttilo, José Julio de Toledo

**Affiliations:** ^1^ Programa de Pós‐Graduação Em Biodiversidade Tropical Universidade Federal Do Amapá (PPGBIO‐UNIFAP) Macapá Brazil; ^2^ Programa Para la Conservación de los Murciélagos de Bolivia (PCMB) Cochabamba Bolivia; ^3^ Instituto de Ecología (INECOL), Red de Ecoetología Xalapa Mexico

**Keywords:** bat flies, bats, host abundance, host importance, host traits, interaction networks, Neotropics

## Abstract

Host functional traits are known to shape host‐parasite interactions across multiple taxa. For bats, ectoparasite loads are often regulated by host body size, diet, longevity, and roosting ecology. Therefore, Neotropical bats represent an ideal system for testing host‐parasite interaction hypotheses, as the second more speciose group of mammals, with a high level of functional diversity. Their primary ectoparasites are highly host‐specific hematophagous bat flies, who spend the most part of their lifetime on their hosts. Here, we evaluated whether bat functional traits influence their importance as hosts within Neotropical bat‐bat fly interactions. We predicted that larger carnivorous, longer‐lived, cavity‐roosting bats would exhibit higher host importance. Using interaction and trait data from published datasets, we constructed a unified interaction network for Neotropical bats and bat flies, calculated network metrics and used generalized additive mixed models to test how host traits define host importance in the network. Trophic level was the only trait with a significant effect, with carnivorous bats significantly less important than both herbivorous and omnivorous species. Although host traits such as body size and roosting behavior are known to influence parasite loads in bats, we suggest that host abundance is the main driver for host importance in the network, since carnivorous bats are the most rare Neotropical species. Therefore, in cases of host decline or local extinction, highly abundant bat species may serve as reservoirs for displaced parasites, promoting their persistence and redistribution within the remaining host community.

## Graphical abstract

We analyzed the influence of bat functional traits on their importance as hosts in bat–bat fly networks. Our results indicate that bat abundance is the main driver for host importance in Neotropical bat–bat fly interactions.

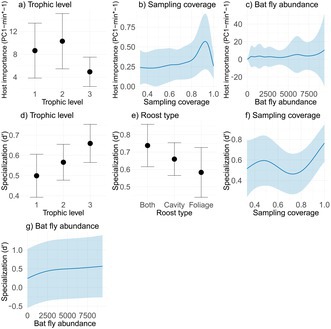

## 1 Introduction

Host–parasite interactions are strongly shaped by the biological traits of both partners. Parasites select hosts that maximize their fitness, often exploiting individuals with traits that facilitate infestation, such as lower grooming efficiency or reduced immunological defenses (Johnson et al. 2019; Manzoli et al. 2021). As a result, most parasites accumulate on few host individuals, producing a highly uneven pattern of parasite loads (Guégan et al. 1992; Patterson et al. 2008). Consequently, hosts with traits that favor parasitism play a disproportionately large role in structuring host–parasite communities (Manzoli et al. 2021).

The relationship between host traits and parasitism is well documented across several taxa. Host body size, for example, is positively associated with parasite richness and biomass, mainly because larger hosts offer more resources, a greater surface area, and reduced competition among parasites (Guégan et al. 1992; Poulin and George‐Nascimento 2007). Longevity may also affect parasite assemblages by extending the temporal window for infestation (Bell and Burt 1991), although evidence is mixed: in fish species, longer‐lived hosts accumulate more parasites, whereas in some mammalian groups, longer‐lived hosts may exhibit lower parasite richness, possibly due to enhanced health or more effective defense mechanisms against infestation (Poulin and Morand 2000; Cooper et al. 2012). Diet shapes parasite exposure too, especially for trophically transmitted endoparasites, with predators more likely to acquire parasites from prey (Luque and Poulin 2004; Poulin and Leung 2011). In ectoparasites, morphological and behavioral adaptations promote niche partitioning, reducing interspecific competition and enhancing survival against host defenses (Bush et al. 2006; Johnson et al. 2012; Hiller et al. 2018).

Bats (Chiroptera) offer an interesting system to examine host–parasite associations due to their unique life history and roosting ecology. They are small‐bodied yet long‐lived mammals with diverse diets ranging from nectar and fruits to insects, vertebrates, and even other bats (Racey and Entwistle 2000; Martínez‐Fonseca et al. 2022). Moreover, many species exhibit plasticity in their social and roosting behavior, switching between solitary and colonial living and using a wide variety of roost types, including foliage, tree cavities, caves, and anthropogenic structures (Lewis 1995; McCracken and Wilkinson 2000). These behavioral shifts influence the opportunities for contact among bats, and between bats and their parasites, with aggregations during the reproductive season potentially enhancing parasite transmission.

Bat flies (Diptera: Streblidae, Nycteribiidae) are obligate hematophagous ectoparasites of bats and display a high degree of host specificity, with most species associated with a single bat host species (Dick and Gettinger 2005; Dick 2007); when multiple host species are used, they usually belong to the same genus or subfamily (Ramírez‐Martínez and Tlapaya‐Romero 2023). Their life cycle depends mainly on the host roost: females deposit third‐instar larvae on the roost substrate, where they pupate and develop until emerging as adults and seek for a host to feed on for the first time (Overal 1980; Meier et al. 1999; Dittmar et al. 2009). Consequently, roosting ecology is a critical determinant of parasite persistence and dispersal. Particularly, cavity and cave roosts provide stable microclimatic conditions that may favor pupal development, increasing bat fly abundance (Poisot et al. 2012), and often, such roosting structures harbor multispecies colonies, potentially increasing opportunities for host‐switching and the origin of new ecological interactions between bat and bat fly species (Dick and Patterson 2007; Patterson et al. 2007). This is further reflected on bat fly prevalence, which is consistently higher in bats species using more protected roosts (ter Hofstede and Fenton 2005; Patterson et al. 2007; Tlapaya‐Romero et al. 2021), and the higher ectoparasite loads harbored by bats that rely on permanent roosts, compared to species that alternate between permanent and ephemeral roosts (ter Hofstede and Fenton 2005; Bolívar‐Cimé et al. 2018).

In host–parasite systems, host‐switching is one of the major drivers of interaction diversity, and similar processes may operate in bat–bat fly interaction networks (de Vienne et al. 2013; Araujo et al. 2015). This phenomenon occurs on an ecological timescale through ecological fitting, whereby parasites exploit novel hosts that fall between their tolerance levels, without requiring prior coevolutionary history and the process can even be facilitated by conditions that increase host contact rates and parasite populations (Janzen 1985; Agosta et al. 2010; Araujo et al. 2015). Although bat flies are usually restricted to a single bat genus or subfamily, some exceptions suggest the occurrence of host‐switching via ecological fitting in this group. This is the case for Neotropical Nycteribiidae, which are typically associated with bats of the family Vespertilionidae (Graciolli et al. 2007), but include *Basilia carteri* and *Basilia mimoni*, species that parasitize phyllostomid bats of the genera Gardnerycteris and Mimon (Graciolli et al. 2007). These bat hosts commonly roost in hollow trees, as do many vespertilionid bats in the Amazon (Aguirre et al. 2003; Bernard and Fenton 2003; Voss et al. 2016), a shared roosting ecology that may have facilitated parasite transfer and subsequent host‐switching. Therefore, even transient interactions can be sufficient to initiate new ecological relationships, particularly in systems characterized by high host mobility and repeated interspecific contact.

In this context, ecological network analysis offers a robust framework for the study of bat–bat fly interactions, with helpful metrics to quantify host species' roles and their contributions to the structure of their ecological networks (Runghen et al. 2021). Evaluating bat species' roles through centrality metrics could provide insights into how individual host species shape network structure, and could help identify hosts with greater potential to participate in host‐switching events (Guimerà and Amaral 2005; Gómez et al. 2013; Llopis‐Belenguer et al. 2020; Piot et al. 2020), as many studies have shown that some species have a disproportionately high network importance and impact on other species interactions, thereby fitting the definition of a highly interactive role (Cruz et al. 2022; Dáttilo et al. 2022; Ratoni et al. 2025). In host–parasite networks, highly interactive hosts are more likely to harbor and transmit parasites, thereby enhancing connectivity within the network, facilitating interactions between species and modules, and maintaining overall parasite diversity (Olesen et al. 2007; Gómez et al. 2013; Piot et al. 2020; Sano et al. 2024). Moreover, highly interactive species participate in more connections within the network, either as primary or temporary hosts, and have a higher probability of acquiring novel parasites and establishing new interactions.

Highly interactive host species also tend to share parasites with multiple other hosts in the network, an attribute that can be measured by closeness and betweenness centrality (Dallas et al. 2019). This reflects the similarity of parasite assemblages among hosts and represents a species‐level measure of connectivity, allowing direct association with host functional traits (Delmas et al. 2019). For instance, Dallas et al. (2019) demonstrated that this host attribute increases with population density and geographic range size in mammals, such as carnivores, ungulates, and primates. Similar patterns were found by Gómez et al. (2013) for primates, and by Cardoso et al. (2021), who reported that host importance increases with host abundance, particularly in frugivorous and omnivorous rodents and marsupials. A similar pattern may occur in bats, as some species exhibit higher bat fly loads during the wet season, a period often associated with increased host abundance and aggregation (Salinas‐Ramos et al. 2017).

Previous studies showed that bats' interactive roles can vary across landscapes (Rivera et al. 2023), reflecting changes in community composition and interaction opportunities. At broader scales, geographically overlapping and roost‐sharing bats tend to share a greater number of ectoparasite species, highlighting the importance of contact opportunities and roost‐mediated transmission (McKee et al. 2019). Accordingly, Dáttilo et al. (2020) reported that proportional range size and body mass enhance host interactivity in bats, analyzing a broad dataset of multiple orders of terrestrial mammals and ectoparasites. Additionally, host abundance and phylogenetic relatedness further shape interaction networks, as more abundant bat species tend to harbor richer bat fly assemblages, and closely related species are more likely to share ectoparasites (Dolabela Falcão et al. 2022). Together, these findings suggest that bat interactive roles in bat‐bat fly networks depend on the combined effects of several bat characteristics, closely linked to bat fly biology, facilitating parasitism.

Although the effects of bat diet and longevity on bat fly parasitism remain poorly understood, both traits may influence host–parasite interactions. Carnivorous and omnivorous bats, for instance, feed on other bats (Esbérard and Bergallo 2004; Oprea et al. 2006), exposing them to their prey's ectoparasites and increasing the likelihood of acquiring novel parasite species. Similarly, Anderson and Sukhdeo (2011) suggested that highly central hosts in food webs tend to harbor richer parasite assemblages. In a recent global assessment, Heckley and Becker (2023) highlighted that the effects of diet and foraging niche are fundamental drivers of host exposure, where non‐frugivore bats exhibit a higher ectoparasite load, although their impact is often mediated by landscape‐scale factors, which can alter host density and encounter rates. However, Luguterah and Lawer (2015) found that insectivorous bats harbor lower ectoparasite loads, potentially due to a more robust immune system and their foraging strategy. As aerial insectivores, they forage without frequent contact with surfaces that may serve as sources of ectoparasite acquisition, thereby reducing opportunities for infestation.

Furthermore, longevity may increase host exposure to ectoparasites by extending the time available for parasite accumulation and transmission (Bell and Burt 1991). In addition, bats experience repeated opportunities for parasite exchange through social interactions such as roost sharing with conspecifics and other species, reproductive aggregations, and in some cases, seasonal migration (Racey 1982; Lewis 1995; McCracken and Wilkinson 2000). Over time these repeated encounters could increase the likelihood of both acquiring a higher richness of ectoparasites and sharing parasite species with other bat hosts (Presley 2004). Longevity is therefore expected to shape parasitism patterns in bats, as longer‐lived species may accumulate exposure to ectoparasites over time, increasing their likelihood of acquiring parasites and forming new host–parasite interactions.

Here, we asked which bat species occupy more interactive roles in Neotropical bat–bat fly interactions, and which host functional traits explain this structural importance and their role as alternative hosts in potential host‐switching events. We integrate two complementary host attributes to establish host importance: (a) parasite species richness, represented by degree centrality; and (b) parasite‐sharing, represented by closeness centrality (measuring a host's proximity to all others in terms of shared parasites), and betweenness centrality (quantifying a host's role in connecting otherwise separate modules; Martín González et al. 2010; Dallas et al. 2019). In bats' particular case, it is difficult to assess the second attribute and search for patterns on it, since bat flies are highly specific and for so, closeness centrality and betweenness centrality values are low (Dáttilo et al. 2020; Bezerra and Bocchiglieri 2023, 2024). This caveat we overcame by combining all interaction data into a single network and combining host attributes into a single measure. Then, we hypothesized that bat traits known to enhance parasitism (body size, longevity, trophic level, and roost type) would also predict their roles as hosts. Specifically, we predicted that larger, carnivorous, longer‐lived, and cavity‐roosting bat species would play a more interactive role in the network. By integrating host trait data with network analyses across Neotropics, our study provides novel insights into the processes shaping host–parasite interactions in this diverse system.

## 2 Methods

### 2.1 Interaction Data

**FIGURE 1:**
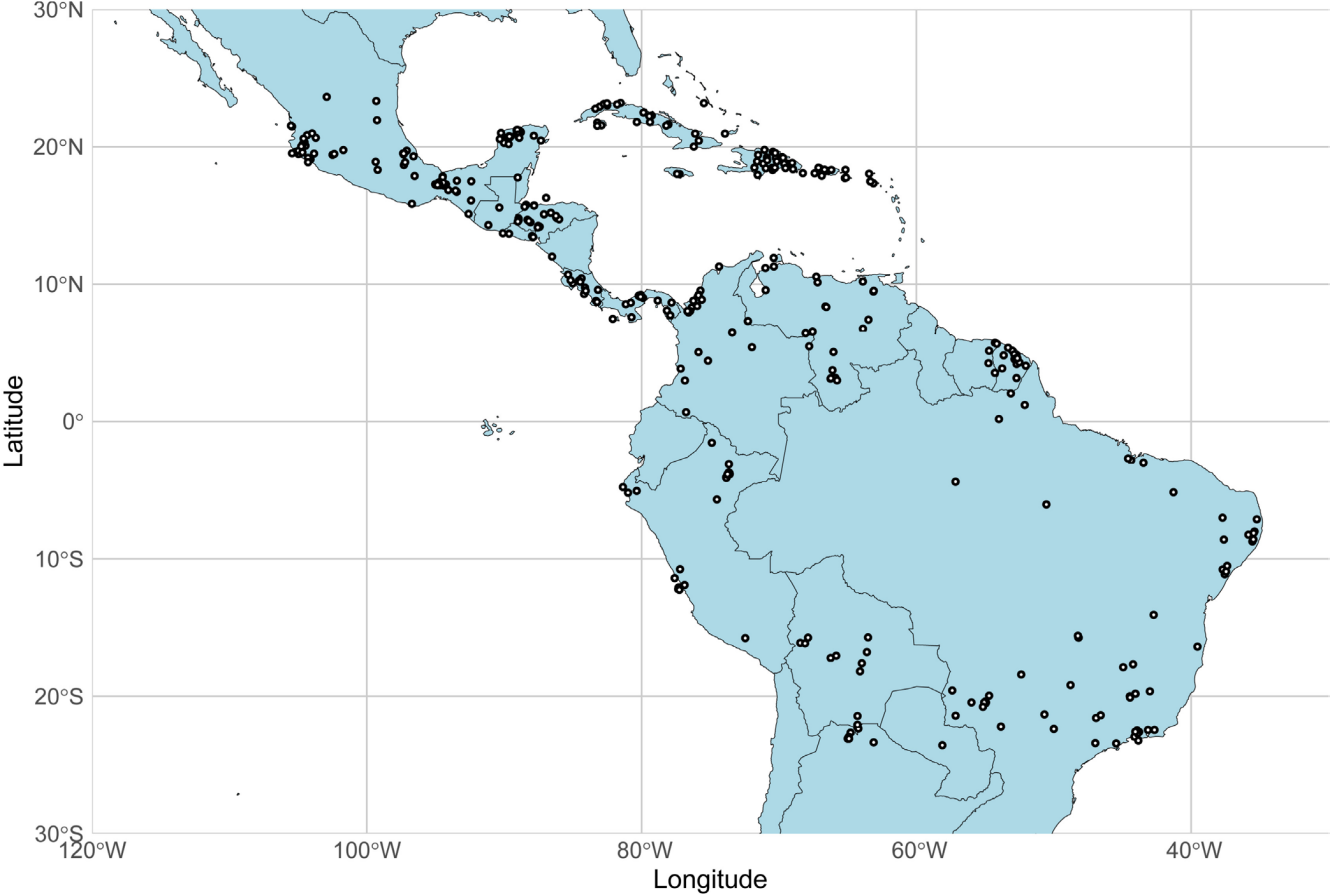
Map illustrating the spatial coverage of sampling efforts for bat–bat fly interaction data in the Neotropics. Each circle represents a unique sampling locality from which data were collected and integrated into the BatFly database (Zapata‐Mesa et al. 2024).

We obtained the interaction data from the database published by Zapata‐Mesa et al. (2024), which contains global‐scale bat–bat fly interactions. To assess bat species importance as hosts in the context of host‐switching processes, we used information on all interactions for each host species in the database, including transient interactions. Accordingly, we consider all interactions to represent co‐occurrence between parasites and hosts, which inherently create opportunities for host‐switching events, including possible human‐mediated transfers (Price et al. 1986; Agosta et al. 2010; Araujo et al. 2015). Then, we filtered the data for the Neotropics, using latitude values (23.5° N, 23.5° S) to create a unified bat–bat fly interaction network at the Neotropical level (Figure 1). We focused on Neotropical bats, since those species share a complex evolutive history, take part in the same ecological processes, and because of their highly morphological and functional diversity (Swartz et al. 2003; Castillo‐Figueroa 2020). We used abundance data for all the calculations and also removed unweighted interactions (presence‐absence data) in order to correctly calculate centrality and sampling coverage.

### 2.2 Functional Trait Data

We obtained information on functional traits from the COMBINE database (Soria et al. 2021) and BatFly database (Zapata‐Mesa et al. 2024). We searched trait data for the bat species we have in our interaction data; then we selected functional traits related to size (adult mass [g]; adult body length [mm]; adult forearm length [mm]), longevity (maximum species longevity [days]), roosting behavior, and trophic level (1: herbivore, 2: omnivore, 3: carnivore). We selected these traits due to their known effect on parasitism by bat flies (see introduction). Most of the traits we used were compiled in the COMBINE database (Soria et al. 2021), which meticulously coalesced data from multiple published sources. They used a dual process of sourcing and imputation to ensure a comprehensive dataset. They obtained the trophic level variable by transforming qualitative, verbal descriptions of species' diets into standardized, semi‐quantitative information, a protocol used in foundational datasets like that by Wilman et al. (2014). Similarly, they gathered data about maximum longevity from a wide range of existing records, and a multiple imputation process was used to fill in missing values.

To assess the effect of roost type on bat importance, we faced two methodological challenges regarding bat roosting behavior and available roosting data. First, because many bat species exhibit behavioral flexibility and can use both cavities and foliage. Second, the structure of the roosting data in the BatFly database (Zapata‐Mesa et al. 2024) consists of a list of roost types for each species, often including multiple roost categories even within the same bat population. Accordingly, we adopted the classification established by ter Hofstede and Fenton (2005), and subsequently used by Bolívar‐Cimé et al. (2018), who grouped bats into three categories (cavity, foliage, or both), thereby facilitating its treatment as a categorical predictor in our linear models.

### 2.3 Data Preparation

Interaction data between bat and bat fly species were compiled from multiple sampling points across the Neotropical region. To assess bat species' importance as hosts for bat flies, we aggregated the records into a single, pooled interaction matrix. In this matrix, we arranged bat species in the rows and bat fly species in the columns. The values within each cell represent the total summed abundance of interactions (the sum of individuals of each fly species in each bat species). All interactions for a single bat species, such as *Carollia perspicillata* , across all sampling points and countries were pooled and entered in its corresponding row. Additionally, since centrality metrics require a fully connected network to be properly calculated, as they are defined within the component to which a species belongs, we excluded from the models those species that were not part of the main component or “giant component” of the network (Brimacombe et al. 2022). Thus, we reduce the risk of biased estimates, such as cases where a species with only a single connection in its own isolated component could otherwise appear to be highly central. A component in a network is a group of species all connected to each other, with no connections outside the group. Thus, the giant component is the largest independent group of connected species in a network (Dorogovtsev et al. 2001).

### 2.4 Data Analysis

Initially, we estimated the ectoparasite sampling coverage for each bat species using the ‘iNext’ package (Chao et al. 2014; Hsieh et al. 2014) in the R platform (R Core Team 2025). Sampling coverage is an estimate of sampling effort using the proportion of ectoparasite species recorded only once for each host (singletons). The coverage calculation is performed assuming that rare species (singletons) are weakly covered by the sampling method (Chao and Jost 2012). Bat species with only a single recorded interaction (i.e., one fly individual) were excluded from subsequent analyses due to insufficient data.

After this, we loaded the data into the “bipartite” package (Dormann et al. 2008, 2009) to calculate three centrality metrics for each bat species:

*Degree (DC)*: The total number of vertices connected to a node, equivalent to the number of species. This measure indicates the intrinsic capacity of a bat species to harbor bat fly species as hosts.

*Closeness (CC)*: The distance (in vertices) of a node relative to others in the network. For a bat host in a bat–bat fly network, this means it belongs to a group of species harboring similar bat fly faunas, or that share species with most of the other host species in the component, which can be due to similarity of functional traits (Dallas et al. 2019).

*Betweenness (BC)*: The extent to which a node acts as a bridge or as a part of the shortest paths between nodes in the network (Dormann et al. 2009; Anderson and Sukhdeo 2011). A bat species with a high level of betweenness could act as a hub of parasites or participate in cross‐species transmission events, connecting otherwise weakly connected groups of species and playing a key role in network architecture.

A higher value for these indices indicates a higher “host importance” (Costa et al. 2007), absorbing and facilitating interactions (i.e., a highly interactive role), by harboring and sharing ectoparasite species (Anderson and Sukhdeo 2011).

We used these three metrics to assess host importance because DC (species richness) alone does not fully capture hosts' interactivity within the network (Dallas et al. 2019). We also summarized the three metrics (DC, CC, and BC) into a single composite axis using Principal Component Analysis (PCA; Vaughan and Ormerod 2005). An approach that reduced redundancy among correlated metrics while retaining their gradient of variation in species' roles (Ramette 2007; Cruz et al. 2022; Dáttilo et al. 2022; Ratoni et al. 2025). Also, this helped us to assess host importance with a single index score, given the low values of CC and BC in this type of network (Dáttilo et al. 2020; Rivera et al. 2023; Bezerra and Bocchiglieri 2023, 2024). The resulting score (PC1), which explained 74.3% of the variability (Figure S1), represented well the three metrics (Square cosine > 0.60 for all; Figure S2), provides an integrated measure of each bat species' importance, which we then correlated to host functional traits to assess how they shape their participation in the bat‐parasite network (Delmas et al. 2019). The obtained PC1 values were negative (Figure S1), which means bats with lower values were more important hosts, so we transformed them by subtracting the minimum value and multiplying by −1, which returned a positive scale.

Furthermore, we use bipartite package (Dormann et al. 2008, 2009) to calculate two additional metrics: network specialization (H2'), and species specialization index (d') for each bat species, as complementary measures. Network specialization (H2') measures how strongly species discriminate among potential interaction partners by contrasting observed interaction patterns with those expected given the structure of the network, ranging from 0 (no specialization) to 1 (perfect specialization; Blüthgen et al. 2006). The species specialization index (d') quantifies the degree of interaction specialization of each species relative to the availability of interaction partners in the network. It is calculated by comparing the observed distribution of interactions of a species with the expected distribution based on the relative abundance of its potential partners, using a standardized Kullback–Leibler distance. Values of d’ range from 0 to 1, where values close to 0 indicate generalized interaction patterns consistent with random partner use, and values close to 1 indicate high specialization (Blüthgen et al. 2006). In the context of bat–bat fly interaction networks, where bats represent the host resource, higher specialization values indicate bat species that interact with a restricted subset of ectoparasites relative to their availability, whereas lower d’ values indicate bat species more likely to be infested by bat flies (i.e., a less restrictive host).

A nonparametric Spearman correlation revealed a weak but significant positive relationship between species specialization (d') and host importance (PC1‐min*‐1) (Rho = 0.15, *p* = 0.035), indicating that highly important host species do not necessarily harbor more generalist assemblages of bat flies.

#### 2.4.1 Construction of the Generalized Additive Mixed Models (GAMM)

To test how bats' functional traits affect their importance as hosts, we use generalized additive mixed models (GAMM) in the “gamm4” R package (Wood and Scheipl 2025). GAMMs are a parametric method that permits the test of nonlinear effects using smoothers, and it permits the inclusion of random effects. After checking the distribution of our data (Figure S3a), we decided to use gamma distribution with a “log” link function, this because gamma distribution works well for continuous, positive, highly right skewed data (Zuur et al. 2009). Then, we constructed our model using all the trait variables we selected. Moreover, to account for the possible effects of sampling intensity, we used sampling coverage and the total number of bat flies found in each bat species as variables in our models, which has been proven to influence the richness of fly species (Lourenço et al. 2016). Because prevalence data and the number of infested individuals were unavailable for several bat species in the database, we did not include mean bat fly abundance per host species in our analyses. Also, to account for unmeasured host‐level heterogeneity and potential taxonomic non‐independence among bat species, we included host genus as a random effect. This approach allows us to control for variation in ecological and behavioral traits that are not explicitly represented by the measured functional traits, while also accounting for trait similarity among closely related species.

We checked in our species' trait data, and all rows with missing (NA) values for any of the predictor variables were removed prior to analysis. Also, to ensure all continuous variables were on a comparable scale, we standardized them to a *z*‐score (a distribution with a mean of 0 and a standard deviation of 1). This standardization process was performed using the “scale()” function in R (R Core Team 2025).

Initially, we used smoothers in all our continuous variables, and after checking their effective degree of freedom (EDF) values (Table S1), we use sampling coverage (EDF = 5.631) and total bat fly abundance (EDF = 7.018) as smooth terms, since all the other variables had an EDF value equal to 1, so the final model syntax was as follows:

Host importance (PC1‐min*‐1) ~ adult mass (g) + max longevity (days) + trophic level (1, 2, 3) + roost (foliage, cavity, both) + adult body length (mm) + adult forearm length (mm) + sampling coverage (smooth term) + fly abundance (smooth term), random (genus).

Then, to evaluate variance inflation in our model, we calculated generalized variance inflation factor, adjusted for their degree of freedom as GVIF^(1/2*DF) with the package “car” of R (Fox et al. 2023). Since all values of GVIF^(1/2*DF) were lower than 2.5 (Table S2), we kept all the variables in our model.

We used the same modeling framework to test the effects of functional traits on species specialization. Specifically, we fitted a GAMM using the “gamm4” package in R (Wood and Scheipl 2025), with specialization as the response variable. Our final model structure went as follows:

Specialization (d') ~ adult mass (g) + max longevity (days) + trophic level (1, 2, 3) + roost (foliage, cavity, both) + adult body length (mm) + adult forearm length (mm; smooth term) + sampling coverage (smooth term) + fly abundance (smooth term), random (genus).

We selected gaussian distribution after graphical inspection (Figure S3b). All predictor variables and random effects, went the same data standardization, handling of missing values, and procedures for evaluating non‐linearity and multicollinearity were identical to those used in the host importance analyses (Table S2). We included host genus as a random effect as well, and we used smoothers form adult forearm length, sampling coverage and fly abundance, after checking for all variables first (Table S1).

## 3 Results

After applying all filters to clean the data, we ended up with 181 bat species, from 8 families: Phyllostomidae (119), Vespertilionidae (24), Mormoopidae (19), Molossidae (8), Emballonuridae (3), Natalidae (5), Furipteridae (1), and Noctilionidae (2). Most of the bat species (121) had data on all the traits we used in our models, and most of them were cavity roosting bats (89), while 15 species were foliage roosting bats, and 17 bat species roost in both types. Data about the values of bats' functional traits can be found in Supporting Information (Table S3).

Host importance (PC1‐min*‐1) went from 0.02 to 11.98 (mean = 1.37; SD = 1.71; Table S4) and the most important species were *Carollia perspicillata* (11.98), *Artibeus jamaicencis* (8.31), and *Artibeus lituratus* (7.35), which means those species are more prone to harbor and share bat fly species (see the list of the 10 most important species in Table S4). From a structural perspective, these hosts function as the primary network hubs, maintaining network structure and improving connectivity. Bat species DC went from 1 to 46 (mean = 6.35; SD = 7.29), with *C. perspicillata* once more as the species with the highest value (46), followed by *A. lituratus* (38) and *A. jamaicencis* (31). As expected, due to the high levels of specificity of bat–bat fly interactions, closeness and betweenness values were very low, with CC going from 0.00003 to 0.001 (mean = 0.0005; SD = 0.0004) while BC went from 0 to 0.217 (mean = 0.007; SD = 0.025).

Network specialization was high (H2' = 0.78), while species specialization (d'), varied within a wide range (mean = 0.60; SD = 0.23), from low (d' = 0.10) to almost perfect specialization (d' = 0.99). The host species with the lower specialization (i.e., bat species with less restricted bat fly assemblages, or higher bat fly colonization susceptibility) were *Sturnira koopmanhilli* (d' = 0.10), *Rinhophylla alethina* (d' = 0.12), and *Anoura latidens* (d' = 0.13).

### 3.1 Generalized Additive Mixed Models

**FIGURE 2:**
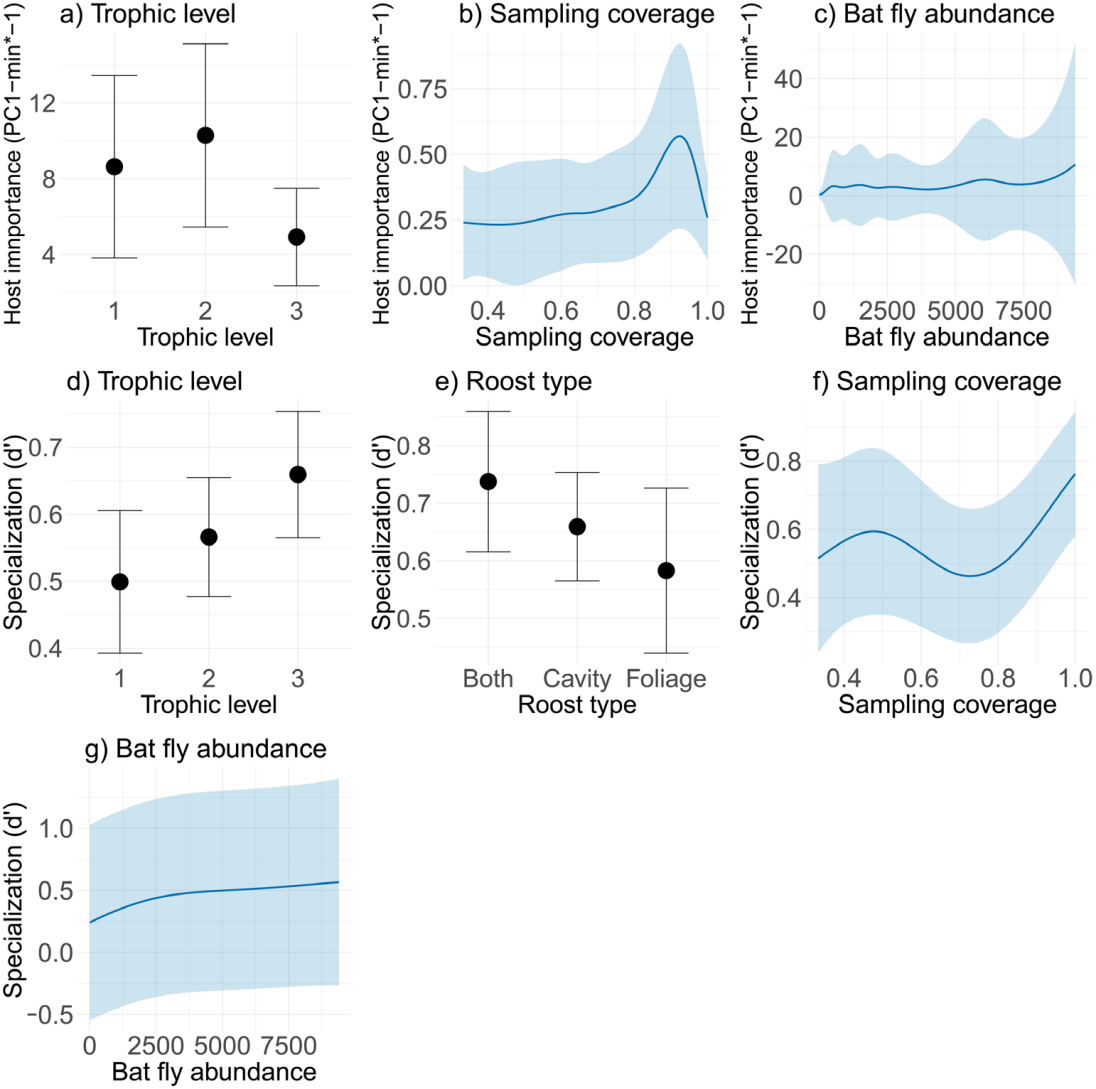
Significative effects on host importancea (PC1‐min*‐1) and host specialization (d') in the Neotropical bat‐bat fly network. Parametric terms (a, d, e), and smooth terms (b, c, f, g).

None of the morphological traits we tested (adult mass, body length, or forearm length) showed a significant effect on host importance, nor did longevity and roost type (all *p* > 0.1; Table 1). Conversely, trophic level showed significant variation (Figure 2a), with carnivorous bats as less important hosts, with values around 0.57 [exp(β)] times lower compared to the other two levels (trophic level 3, *t* = −3.11, *p* = 0.002; Table 2). We also found a significant effect from the smooth terms, with host importance increasing with sampling coverage, up to approximately 90% and then a decline toward full coverage (EDF = 4.858, *F* = 5.366, *p* = 0.001; Figure 2b). Similarly, the total bat fly abundance exhibited a positive effect, with a gradual increase across higher abundance values (EDF = 7.575, *F* = 32.387, *p* < 0.001; Figure 2c). Overall, the model explained about 82% of the adjusted variance, with genus as a random effect contributing slightly (Variance = 0.12, SD = 0.35), which means a moderate taxonomic signal.

**TABLE 1:** Effect of the parametric and smooth terms on the generalized additive mixed effect models (GAMM) for host importance (PC1‐min*‐1).

Parametric terms	*β*	T	*p*
Adult mass (g)	−0.040	−0.472	0.638
Max longevity (days)	0.090	1.537	0.127
Trophic level (2)	0.170	1.021	0.310
Trophic level (3)	**−0.561**	**−3.110**	**0.002**
Roost (cavity)	0.020	0.113	0.911
Roost (foliage)	−0.194	−0.913	0.363
Adult body length (mm)	−0.127	−1.399	0.165
Adult forearm length (mm)	0.161	1.463	0.147

Smooth terms	EDF	*F*	*p*
Sampling coverage	**4.847**	**5.328**	**0.001**
Fly abundance	**7.575**	**32.387**	**< 0.001**
R‐sq = 0.818			

*Note:* Values in bold show significant effects (*p* < 0.05).

Abbreviation: EDF, effective degree of freedom.

**TABLE 2:** Effect of the parametric and smooth terms on the generalized additive mixed effect models (GAMM) for host specialization (d').

Parametric terms	*β*	T	*p*
Adult mass (g)	−0.007	−0.276	0.783
Max longevity (days)	−0.029	−1.718	0.089
Trophic level (2)	0.067	1.403	0.164
Trophic level (3)	**0.160**	**3.066**	**0.003**
Roost (cavity)	−0.078	−1.561	0.121
Roost (foliage)	**−0.155**	**−2.485**	**0.015**
Adult body length (mm)	0.027	1.000	0.319

Smooth terms	EDF	*F*	*p*
Adult forearm length (mm)	1.661	0.553	0.407
Sampling coverage	**3.233**	**12.644**	**< 0.001**
Fly abundance	**2.178**	**7.519**	**0.001**
R‐sq = 0.421			

*Note:* Values in bold show significant effects (*p* < 0.05).

Abbreviation: EDF, effective degree of freedom.

Bats' trophic level also has a significant effect on bat species specialization (Table 2), with higher specialization in carnivorous bats (Figure 2d), around 1.17 [exp(β)] times higher (trophic level 3, *t* = 3.066, *p* = 0.003) than in the other two levels. Also, foliage roosting bats were around 0.86 [exp(β)] times less specialized than the other two roosting categories (foliage roosting bats, *t* = −2.485, *p* = 0.015; Figure 2e). Sampling coverage and bat fly abundance (both smooth terms) also affected specialization (Table 2), with a non‐linear response to sampling coverage, increasing up to approximately 20%, declining until around 60%, and then rising sharply toward higher sampling coverage levels (EDF = 3.233, F = 12.644, *p* < 0.001; Figure 2f). Specialization also increased with total fly abundance, showing a steep initial rise at low abundances (up to ~2500 individuals) followed by a sustained increase as abundance continued to grow (Figure 2g). The model explained around 42% of the adjusted variance, and the influence of host genus was lower than for the host importance model (Variance = 0.12, SD = 0.35), indicating a lower influence of host taxonomy in this host property.

## 4 Discussion

We provide a comprehensive assessment of bats' importance in bat‐bat fly networks in the Neotropical region, focusing on their role as hosts and the influence of host functional traits. Network specialization was high, consistent with the known structure of bat–bat fly interaction networks (Saldaña‐Vázquez et al. 2019). After testing for the relationship between host importance and specialization, we noted a slight positive relationship, indicating that the most important species do not necessarily require a generalist fly assemblage to be highly interactive, but rather it might be more important for them to evenly share ectoparasites with other host species (Dallas et al. 2019). In fact, the most important species exhibited high specialization values as well ( *Carollia perspicillata* = 0.83, *Artibeus jamaicensis* = 0.66, *Artibeus lituratus* = 0.73; Table S4). We initially hypothesized that host functional traits associated with a higher parasite burden would be associated with higher host importance and, consequently, a more interactive role in bat–bat fly networks. Contrary to most of our expectations, only trophic level significantly influenced host importance, and its effect was opposite to our initial predictions, with carnivorous bats found as more peripheral species, and also the most specialized. We also detected a subtle variation in host importance and specialization across bat genera.

### 4.1 Effects of Diet on Host Importance and Specialization

Contrary to our predictions, carnivorous bats were the less important hosts in the network and exhibited the highest levels of specialization. This pattern may be explained by a combination of ecological factors, beginning with host abundance. In the Neotropics, carnivorous bats tend to be less abundant than plant‐feeding species, likely due to the higher and more predictable availability of energy in plant‐based resources (Arita 1993). Host abundance is closely linked to population density, which strongly influences parasite transmission rates (Arneberg et al. 1998; Vázquez et al. 2005). In bats, more abundant species are known to harbor richer ectoparasite assemblages (Luguterah and Lawer 2015; Lourenço et al. 2016; Dolabela Falcão et al. 2022; Saldaña‐Vázquez et al. 2024), and higher abundance not only increases DC by promoting higher bat fly species richness, but also CC and BC (Dáttilo et al. 2020; Bezerra and Bocchiglieri 2023, 2024), by facilitating parasite sharing among host species through increased encounter rates. Such encounters are particularly critical for bat fly transmission, which occurs primarily during roost sharing, especially when newly emerged flies actively move between hosts in search of new resources (Dittmar et al. 2009). Conversely, the lower abundance of carnivorous bats likely limits both parasite diversity and opportunities for horizontal transmission between bat species. Additionally, Luguterah and Lawer (2015) suggest that carnivorous bats may possess more robust immune defenses associated with their diet, potentially further reducing new parasite species establishment. Although a high‐protein diet can enhance immune function, it is also energetically costly, whit omnivorous species, with a more balanced intake of proteins, carbohydrates, fats, and micronutrients, may therefore exhibit more efficient immune responses (Vestey et al. 1993). However, this pattern was not supported by our results, as omnivorous bats were neither less important nor more specialized than other trophic groups. Consequently, variation in immune investment associated with diet composition is unlikely to be a driver of host importance or specialization in bat–bat fly interaction networks.

Sampling‐related factors may also contribute to this pattern. Carnivorous bats, including strictly insectivorous species, generally possess more efficient echolocation systems, reducing capture success with mist nets and harp traps (Meyer et al. 2011). Such biases may lead to an underrepresentation of their ectoparasite fauna. This interpretation is further supported by our results, which show that host importance increases with sampling coverage (up to around 90%–Figure 2b) as well as with total fly abundance (Figure 2c).

Together, these factors likely place carnivorous bats in more peripheral positions within bat‐bat fly networks, with a low participation in network connectivity, and characterizing them as restricted, less permissive hosts that are more unlikely to participate in host‐switching processes. However, this pattern raises an apparent contradiction, as the documented cases of potential host‐switching we mention previously, involve the genera *Mimon* and *Gardnerycteris*, both presented as carnivorous bats (trophic level = 3) in the database. This discrepancy may be explained by the taxonomic identity of the ectoparasites involved: the bat flies associated with these bats belong to the family Nycteribiidae, which generally exhibits lower host specificity than Streblidae (Presley 2011). Thus, in such cases, host‐switching may have required only the opportunity for contact with a compatible host, following a decline in the abundance of the original host (Manzoli et al. 2021; Farrell et al. 2021). Although bat flies are generally highly host‐specific ectoparasites, some species may have a greater capacity for host‐switching under conditions where opportunity and need coincide (D'Bastiani et al. 2020; Manzoli et al. 2021). Empirical evidence shows that bat flies are highly efficient at locating their main hosts in their roosts, with the confirmation of experimental data, and that many species starve in the absence of a suitable host (Overal 1980). Nevertheless, it is unlikely that strict host specificity represents an absolute rule for all bat fly species, as parasites usually exhibit a high level of adaptability (Cruz‐Laufer et al. 2025), which may allow certain lineages to persist through host‐switching when environmental or host demographic conditions favor such transitions.

Interestingly, despite the overall positive correlation between host importance and specialization, diet showed opposite effects on these two metrics. This suggests that dietary‐related conditions (mentioned above) may constrain bat–bat fly associations, resulting in more restricted parasite assemblages as well, with carnivorous bats functioning as more selective hosts, interacting with few parasite species that are not common in the network.

### 4.2 Effects of Host Roosting Behavior

Bat specialization was also influenced by roosting behavior, with foliage‐roosting bats exhibiting the lowest specialization values. At first glance, this result may seem counterintuitive, given that foliage‐roosting species generally show lower ectoparasite prevalence and harbor fly assemblages with reduced species richness (ter Hofstede and Fenton 2005; Patterson et al. 2007), mainly because such roosting behavior expose bat flies to unfavorable external weather conditions (Poisot et al. 2012; Salinas‐Ramos et al. 2018). However, low specialization does not necessarily require a high number of interactions. Species with few interaction partners and low parasite abundance may still exhibit low specialization values. In such cases, the typically low parasite loads found in these bats contribute to a more even proportional distribution among parasite species (i.e., all bat fly species are in similar low abundances), which directly affects the calculation of specialization. As a result, hosts specialization is low, particularly when their limited interactions involve the most widespread and abundant bat fly species in the network, reflecting minimal deviation from a scenario of random partner use (Blüthgen et al. 2006). Likewise, most foliage‐roosting bats are frugivorous phyllostomids (Garbino and Tavares 2018), particularly from the subfamilies Stenodermatinae (*Artibeus, Dermanura, Centurio, Mesophylla, Platyrrhinus, Sturnira*, and *Vampyrodes*) and Carollinae (*Rhinophylla*), which comprise some of the most abundant and widespread bat groups in the Neotropics (Arita 1993). Thus, foliage‐roosting species tend to share bat fly species with highly important hosts such as *Carollia perspicillata, Artibeus jamaicensis*, and *Artibeus lituratus* . In particular, *A. jamaicensis* could act as an ecological bridge, given its ability to roost in both cavities and foliage, which allows it to serve as a hub for ectoparasite exchange across otherwise distinct roosting communities. These important bat species, in turn, concentrate a large number of interactions on a small subset of primary bat fly species, resulting in a high proportion of interactions directed toward few partners and, consequently, higher specialization values (Blüthgen et al. 2006).

The negative effect of host roosting behavior on specialization, coupled with the absence of a significant relationship with host importance, is somewhat unexpected, given that host roosting ecology is often considered one of the most influential factors shaping bat–bat fly interactions. One possible explanation relates to the use of large and stable shelters (such as caves, abandoned buildings, and grottos) by dense aggregations of multiple bat species. In these environments, bat flies may encounter a high availability of “high‐quality” hosts (i.e., their primary hosts; Manzoli et al. 2021), constraining their use of alternative host species to closely related hosts when available. This interpretation is consistent with the known effect of host phylogeny on parasite sharing, as ectoparasite exchange among cave‐roosting bats is more frequent among closely related species (McKee et al. 2019), and that roost selection can also have a phylogenetic influence (Betke et al. 2024). Consequently, interactions may remain confined within compact, phylogenetically structured host groups, resulting in the formation of peripheral modules.

Working with bat roosting data entails several inherent complexities. Roosting behavior in Neotropical bats is highly variable, both within and among species, as reflected in the structure of our dataset (see Methods). Consequently, assigning a single “primary” roost type to each species, particularly when aiming for a more precise quantitative classification (e.g., Patterson et al. 2007; Hiller et al. 2018), is challenging. This difficulty persists even in studies specifically designed to identify principal roost types (Voss et al. 2016).

While the categorical approach adopted here captures major ecological differences among roost types, a more standardized and quantitative framework would likely enhance future analyses. The development of structured, comprehensive databases on roost use, incorporating detailed field data on roost sharing, would enable the construction of ecologically meaningful roosting guilds (see Voss et al. 2016). Such advances would strengthen macroecological investigations and potentially provide more refined insights into how roosting ecology shapes bat–bat fly interaction networks.

### 4.3 Sampling Completeness and Bat Fly Abundance

Both host importance and specialization were affected by sampling completeness and bat fly abundance and this may be linked to sampling intensity. Interactions are usually harder to record than species (Poisot et al. 2012; Jordano 2016), therefore, higher sampling intensity could increase the observed host interactions by enhancing the detection, reducing the number of missing links in the network, leading to more accurate estimates of direct connections (DC) and also detecting shared parasite species (CC and BC; Dallas et al. 2019).

The effect of sampling intensity on specialization is also directly linked to the proportional distribution of interactions. As sampling effort increases, rare interactions are more frequently detected; however, these interactions typically represent a small fraction relative to the dominant interactions with a host's primary bat fly species. Consequently, the proportional weight of rare interactions remains low, which directly influences the calculation of Blüthgen's specialization (Blüthgen et al. 2006).

Increased sampling effort also raises the likelihood of recording transient interactions and contamination events, particularly for frequently sampled host species (Dick 2007). In the context of this study, where we aim to evaluate bat species as potential hosts in host‐switching processes, such transient interactions (whether naturally occurring or human‐mediated), are relevant components of parasite transmission dynamics (Price et al. 1986), as humans are well‐documented vectors of parasite spread among wildlife (Reynolds and Barton 2013; Ballmann et al. 2017), sometimes with dire consequences, as exemplified by white‐nose syndrome in bats and chytridiomycosis in anurans, both of which can be transmitted via clothing and field equipment (Reynolds and Barton 2013; Frick et al. 2016; Ballmann et al. 2017; Schilliger et al. 2023).

Although successful host‐switching in bat flies requires more than contact alone (specifically, the establishment of a reproductively viable parasite population; Dick and Patterson 2007), frequent handling and monitoring of bats make it necessary to consider these human‐mediated contacts when interpreting interaction data (Price et al. 1986). Given that bats are a highly surveyed mammal group, due to their value as bioindicators, acknowledging the potential contribution of transient and contamination‐driven interactions is essential for a comprehensive understanding of bat–bat fly interactions.

### 4.4 Host Size and Longevity

Regarding host size, previous studies have shown that larger bats harbor richer and more abundant bat fly assemblages (Patterson et al. 2008), while bat closeness centrality appears to increase with bats' body weight too in networks composed of several terrestrial mammals and ectoparasites (Dáttilo et al. 2020). Our results demonstrate that although some host traits boost higher parasite richness (DC), that does not translate into higher host interactivity in the network (Dallas et al. 2019). Here, we found no significant effect of host body size on host importance. This means a host species may support many parasite species yet remain relatively isolated in the network (forming its own module) if it does not share parasite species with other hosts. This could be related to hosts' ecological and phylogenetic distinctness in the network (as we discussed previously); therefore, host phylogenetic distinctness among the network is needed to address in future analyses (Runghen et al. 2021). Nonetheless, our results show now that host body size does not influence bats' interactive role in bat–bat fly networks.

Regarding longevity, our assumption that bats could accumulate parasite species throughout their lives was incorrect. Longevity and size are usually strongly correlated variables, so when analyzing longevity separately, it is necessary to mathematically correct for the effect of size (Cooper et al. 2012). Bats far exceed the longevity levels of other mammals of the same size (Wilkinson and South 2002), and for this reason, we can state that the parasite accumulation effect (Bell and Burt 1991) does not apply to these mammals, and therefore, longevity does not affect their interactions with their ectoparasitic flies. Other factors could also influence our results, such as levels of immunocompetence and grooming behaviors, which could impact parasite abundance, richness, and sharing among different bat species (Overal 1980; ter Hofstede and Fenton 2005).

## 5 CONCLUSIONS

Overall, host abundance emerges as a primary determinant of host importance in Neotropical bat–bat fly interactions, with the most abundant bat species occupying the most influential network positions. High host abundance not only increases bat fly species richness but also enhances ectoparasite transmission dynamics. Consequently, abundant bats are more likely to function as hubs within these networks and represent the most probable targets for host‐switching events. Changes in host abundance patterns are therefore expected to alter species roles within bat‐bat fly networks, promoting abundant species to central positions while relegating rarer hosts to more peripheral roles. Moreover, in scenarios of host population decline or local extinction, abundant bat species may act as reservoirs for parasites formerly associated with lost hosts, potentially facilitating parasite persistence and redistribution within the remaining host community.

## Author Contributions

**Paulo Mejia:** conceptualization (equal), data curation (lead), formal analysis (lead), funding acquisition (equal), investigation (equal), methodology (lead), project administration (equal), supervision (equal), validation (equal), visualization (lead), writing – original draft (lead), writing – review and editing (lead). **Wesley Dáttilo:** formal analysis (equal), investigation (equal), methodology (equal), project administration (equal), supervision (equal), validation (equal), writing – original draft (equal), writing – review and editing (equal). **José Julio de Toledo:** conceptualization (equal), formal analysis (equal), funding acquisition (equal), investigation (equal), methodology (equal), project administration (lead), supervision (equal), validation (equal), writing – original draft (equal), writing – review and editing (equal).

## Funding

This work was supported by Conselho Nacional de Desenvolvimento Científico e Tecnológico (CNPq): 459735/2014‐4, 316281/2021‐2, 409827/2021‐5, 444350/2024‐1, 312930/2025‐9, 381743/2026‐8. Coordenação de Aperfeiçoamento de Pessoal de Nível Superior (CAPES), 88887.662021/2022‐00.

## Conflicts of Interest

The authors declare no conflicts of interest.

## Supporting information


**Table S1:** Effective degree of freedom (EDF) of all numerical variables tested initially with smoothers in the generalized mixed effect models (GAMM).
**Table S2:** Calculated values of the generalized variance inflation factor, adjusted for their degree of freedom, resulting from a generalized linear model (GLM) fitted with the variables examined.
**Table S3:** Summary information on the functional traits of the bat species examined.
**Table S4:** List of species included in our GAMMs, ranked by host importance and with their species specialization values.
**Figure S1:** Principal Component Analysis (PCA) of bat species centrality metrics in bat–bat fly interaction networks. The biplot shows the loadings of degree, closeness, and betweenness centrality on the first two principal components. Dim1 explains 74.3% of the total variance and represents a general gradient of host importance, with all three centrality measures loading strongly and in the same direction. Dim2 explains 20.5% of the variance.
**Figure S2:** Quality of representation (squared cosine) of each centrality metric on the first principal component (PC1) of the PCA. Higher cos2 values indicate that a larger proportion of the variance of a given metric is captured by PC1. Degree centrality shows the highest contribution to PC1, followed by betweenness and closeness, confirming that PC1 primarily represents overall host importance in bat–bat fly interaction networks.
**Figure S3:** Density distributions of host importance (PC1‐min*‐1) and specialization (d) metrics used to guide the choice of error distributions in generalized additive mixed models testing the effects of host functional traits.

## Data Availability

The data supporting the findings of this study are openly available in Figshare at https://doi.org/10.6084/m9.figshare.31342597. All raw data on bat–bat fly interactions and host functional traits were previously published in the following data papers: Zapata‐Mesa et al. (2024; https://doi.org/10.5281/zenodo.10019756) and Soria et al. (2021; https://doi.org/10.6084/m9.figshare.13028255.v4).
