# Supplementary material for: Host Abundance Predicts Interactive Roles in Neotropical Bat–Bat Fly Interactions
Source: Ecol Evol. 2026 May 13;16(5):e73640. doi: 10.1002/ece3.73640 (PMC13172273; doi:10.1002/ece3.73640)
Supplement: Supplementary file 1 — Table S1: Effective degree of freedom (EDF) of all numerical variables tested initially with smoothers in the generalized mixed effect models (GAMM). Table S2: Calculated values of the generalized variance inflation factor, adjusted for their degree of freedom, resulting from a generalized linear model (GLM) fitted with the variables examined. Table S3: Summary information on the functional traits of the bat species examined. Table S4: List of species included in our GAMMs, ranked by host importance and with their species specialization values. Figure S1: Principal Component Analysis (PCA) of bat species centrality metrics in bat–bat fly interaction networks. The biplot shows the loadings of degree, closeness, and betweenness centrality on the first two principal components. Dim1 explains 74.3% of the total variance and represents a general gradient of host importance, with all three centrality measures loading strongly and in the same direction. Dim2 explains 20.5% of the variance. Figure S2: Quality of representation (squared cosine) of each centrality metric on the first principal component (PC1) of the PCA. Higher cos2 values indicate that a larger proportion of the variance of a given metric is captured by PC1. Degree centrality shows the highest contribution to PC1, followed by betweenness and closeness, confirming that PC1 primarily represents overall host importance in bat–bat fly interaction networks. Figure S3: Density distributions of host importance (PC1‐min*‐1) and specialization (d) metrics used to guide the choice of error distributions in generalized additive mixed models testing the effects of host functional traits. [file ECE3-16-e73640-s001.docx]

SUPPORTING INFORMATION FOR THE MANUSCRIPT INTITULATED: Host abundance predicts interactive roles in Neotropical bat–bat fly interactions.

TABLES

Table S1. Effective degree of freedom (EDF) of all numerical variables tested initially with smoothers in the generalized mixed effect models (GAMM).

| VARIABLE | EDF HOST IMPORTANCE MODEL | EDF SPECIALIZATION MODEL |
| --- | --- | --- |
| Adult mass (g) | 1.001 | 1 |
| Max longevity (days) | 1.001 | 1 |
| Adult body length (mm) | 1 | 1 |
| Adult forearm length (mm) | 1 | 1.661 |
| Sampling coverage | 5.631 | 3.233 |
| Bat fly abundance | 7.018 | 2.177 |

Variables with EDF=~1 values were later used as parametric effects in our models.

Table S2. Calculated values of the generalized variance inflation factor, adjusted for their degree of freedom, resulting from a generalized linear model (GLM) fitted with the variables examined.

| VARIABLE | GVIF | Df | GVIF^(1/(2*Df)) |
| --- | --- | --- | --- |
| Adult mass (g) | 2.88 | 1.00 | 1.70 |
| Max longevity (days) | 1.16 | 1.00 | 1.08 |
| Trophic level (1, 2, 3) | 1.33 | 2.00 | 1.07 |
| Roost (foliage, cavity, both) | 1.55 | 2.00 | 1.12 |
| Adult body length (mm) | 2.99 | 1.00 | 1.73 |
| Adult forearm length (mm) | 4.11 | 1.00 | 2.03 |
| Sampling coverage | 1.16 | 1.00 | 1.08 |
| Fly abundance | 1.24 | 1.00 | 1.11 |

GVIF^(1/(2*Df)) values apply for both models (host importance and specialization).

Table S3. Summary information on the functional traits of the bat species examined.

| FUNCTIONAL TRAITS | MAX | MIN | MEAN | SD |
| --- | --- | --- | --- | --- |
| Adult mass (g) | 134 | 3.15 | 19.58 | 17.48 |
| Adult body lenght (mm) | 128.5 | 43 | 70.63 | 14.98 |
| Adult forearm (mm) | 83.27 | 28.49 | 45.64 | 10.56 |
| Max longevity (days) | 10658 | 939.88 | 5267.28 | 1361.70 |

Table S4. Listo of species included in our GAMMs, ranked by host importance and with their species specialization values.

| Host species | Host importance (PC1-min*-1) | Host specialization (d') |
| --- | --- | --- |
| *Carollia perspicillata* | 11.98 | 0.83 |
| *Artibeus jamaicensis* | 8.31 | 0.67 |
| *Artibeus lituratus* | 7.35 | 0.73 |
| *Desmodus rotundus* | 5.92 | 0.89 |
| *Glossophaga soricina* | 4.35 | 0.58 |
| *Pteronotus mesoamericanus* | 4.10 | 0.59 |
| *Sturnira lilium* | 3.98 | 0.78 |
| *Artibeus planirostris* | 3.74 | 0.69 |
| *Sturnira hondurensis* | 3.60 | 0.68 |
| *Phyllostomus hastatus* | 3.49 | 0.87 |
| *Carollia brevicauda* | 3.19 | 0.54 |
| *Platyrrhinus lineatus* | 3.06 | 0.74 |
| *Phyllostomus discolor* | 3.02 | 0.93 |
| *Sturnira parvidens* | 2.93 | 0.61 |
| *Anoura geoffroyi* | 2.89 | 0.99 |
| *Leptonycteris yerbabuenae* | 2.88 | 0.64 |
| *Phyllostomus elongatus* | 2.80 | 0.58 |
| *Carollia sowelli* | 2.80 | 0.35 |
| *Lophostoma silvicolum* | 2.79 | 0.63 |
| *Trachops cirrhosus* | 2.55 | 0.89 |
| *Noctilio albiventris* | 2.51 | 0.94 |
| *Lophostoma carrikeri* | 2.46 | 0.85 |
| *Diphylla ecaudata* | 2.26 | 0.77 |
| *Lophostoma brasiliense* | 2.22 | 0.62 |
| *Anoura caudifer* | 2.14 | 0.73 |
| *Dermanura cinerea* | 2.02 | 0.21 |
| *Leptonycteris curasoae* | 1.94 | 0.86 |
| *Phyllonycteris poeyi* | 1.93 | 0.78 |
| *Carollia castanea* | 1.91 | 0.35 |
| *Diaemus youngi* | 1.80 | 0.96 |
| *Lonchophylla mordax* | 1.79 | 0.82 |
| *Mimon cozumelae* | 1.75 | 0.51 |
| *Lonchophylla robusta* | 1.62 | 0.81 |
| *Myotis riparius* | 1.58 | 0.77 |
| *Macrotus waterhousii* | 1.54 | 0.85 |
| *Glossophaga commissarisi* | 1.50 | 0.42 |
| *Dermanura phaeotis* | 1.47 | 0.63 |
| *Pteronotus rubiginosus* | 1.46 | 0.66 |
| *Monophyllus redmani* | 1.42 | 0.85 |
| *Micronycteris minuta* | 1.30 | 0.57 |
| *Platyrrhinus helleri* | 1.29 | 0.52 |
| *Erophylla bombifrons* | 1.28 | 0.76 |
| *Dermanura tolteca* | 1.27 | 0.54 |
| *Mormoops megalophylla* | 1.22 | 0.58 |
| *Lonchophylla thomasi* | 1.20 | 0.67 |
| *Myotis albescens* | 1.18 | 0.93 |
| *Glyphonycteris daviesi* | 1.13 | 0.28 |
| *Lampronycteris brachyotis* | 1.13 | 0.29 |
| *Gardnerycteris crenulatum* | 1.12 | 0.95 |
| *Lonchorhina aurita* | 1.11 | 0.88 |
| *Natalus mexicanus* | 1.08 | 0.86 |
| *Brachyphylla nana* | 1.07 | 0.73 |
| *Sturnira tildae* | 1.04 | 0.31 |
| *Pteronotus gymnonotus* | 1.04 | 0.85 |
| *Micronycteris megalotis* | 1.03 | 0.40 |
| *Brachyphylla cavernarum* | 0.98 | 0.75 |
| *Leptonycteris nivalis* | 0.88 | 0.56 |
| *Pteronotus personatus* | 0.86 | 0.50 |
| *Trinycteris nicefori* | 0.84 | 0.97 |
| *Pteronotus quadridens* | 0.84 | 0.53 |
| *Artibeus inopinatus* | 0.82 | 0.50 |
| *Choeronycteris mexicana* | 0.79 | 0.55 |
| *Erophylla sezekorni* | 0.79 | 0.60 |
| *Chiroderma doriae* | 0.78 | 0.26 |
| *Chrotopterus auritus* | 0.78 | 0.83 |
| *Eptesicus furinalis* | 0.77 | 0.86 |
| *Lonchophylla dekeyseri* | 0.74 | 0.44 |
| *Eptesicus brasiliensis* | 0.71 | 0.81 |
| *Platyrrhinus vittatus* | 0.68 | 0.47 |
| *Rhinophylla pumilio* | 0.65 | 0.77 |
| *Dermanura rava* | 0.62 | 0.85 |
| *Vampyressa thyone* | 0.60 | 0.77 |
| *Platyrrhinus dorsalis* | 0.59 | 0.27 |
| *Enchisthenes hartii* | 0.58 | 0.37 |
| *Platalina genovensium* | 0.58 | 0.52 |
| *Artibeus aequatorialis* | 0.53 | 0.32 |
| *Carollia subrufa* | 0.50 | 0.20 |
| *Chiroderma villosum* | 0.48 | 0.47 |
| *Artibeus hirsutus* | 0.46 | 0.29 |
| *Saccopteryx bilineata* | 0.43 | 0.65 |
| *Mimon bennettii* | 0.43 | 0.39 |
| *Pteronotus parnellii* | 0.43 | 0.57 |
| *Lophostoma evotis* | 0.43 | 0.59 |
| *Myotis ruber* | 0.42 | 0.94 |
| *Hylonycteris underwoodi* | 0.41 | 0.65 |
| *Glossophaga morenoi* | 0.41 | 0.35 |
| *Sturnira ludovici* | 0.41 | 0.17 |
| *Tonatia bidens* | 0.40 | 0.42 |
| *Myotis keaysi* | 0.39 | 0.61 |
| *Rhinophylla fischerae* | 0.36 | 0.61 |
| *Molossops temminckii* | 0.35 | 0.32 |
| *Micronycteris schmidtorum* | 0.34 | 0.50 |
| *Platyrrhinus brachycephalus* | 0.32 | 0.24 |
| *Centurio senex* | 0.32 | 0.16 |
| *Lonchophylla peracchii* | 0.32 | 0.48 |
| *Natalus tumidirostris* | 0.29 | 0.49 |
| *Vampyrodes major* | 0.28 | 0.14 |
| *Mesophylla macconnelli* | 0.28 | 0.76 |
| *Phyllostomus latifolius* | 0.28 | 0.39 |
| *Myotis elegans* | 0.27 | 0.82 |
| *Furipterus horrens* | 0.23 | 0.80 |
| *Pteronotus macleayii* | 0.23 | 0.54 |
| *Myotis velifer* | 0.22 | 0.51 |
| *Rhynchonycteris naso* | 0.19 | 0.33 |
| *Platyrrhinus recifinus* | 0.19 | 0.21 |
| *Anoura latidens* | 0.19 | 0.13 |
| *Sturnira erythromos* | 0.15 | 0.41 |
| *Eptesicus fuscus* | 0.15 | 0.29 |
| *Rhinophylla alethina* | 0.15 | 0.12 |
| *Sturnira magna* | 0.15 | 0.35 |
| *Balantiopteryx plicata* | 0.15 | 0.47 |
| *Vampyressa pusilla* | 0.12 | 0.72 |
| *Chiroderma salvini* | 0.12 | 0.80 |
| *Uroderma bilobatum* | 0.12 | 0.33 |
| *Histiotus velatus* | 0.12 | 0.75 |
| *Pteronotus davyi* | 0.09 | 0.79 |
| *Eptesicus diminutus* | 0.08 | 0.66 |
| *Nycticeius humeralis* | 0.08 | 0.66 |
| *Lasiurus borealis* | 0.08 | 0.92 |
| *Tadarida brasiliensis* | 0.05 | 0.56 |
| *Molossus molossus* | 0.02 | 0.82 |

FIGURES


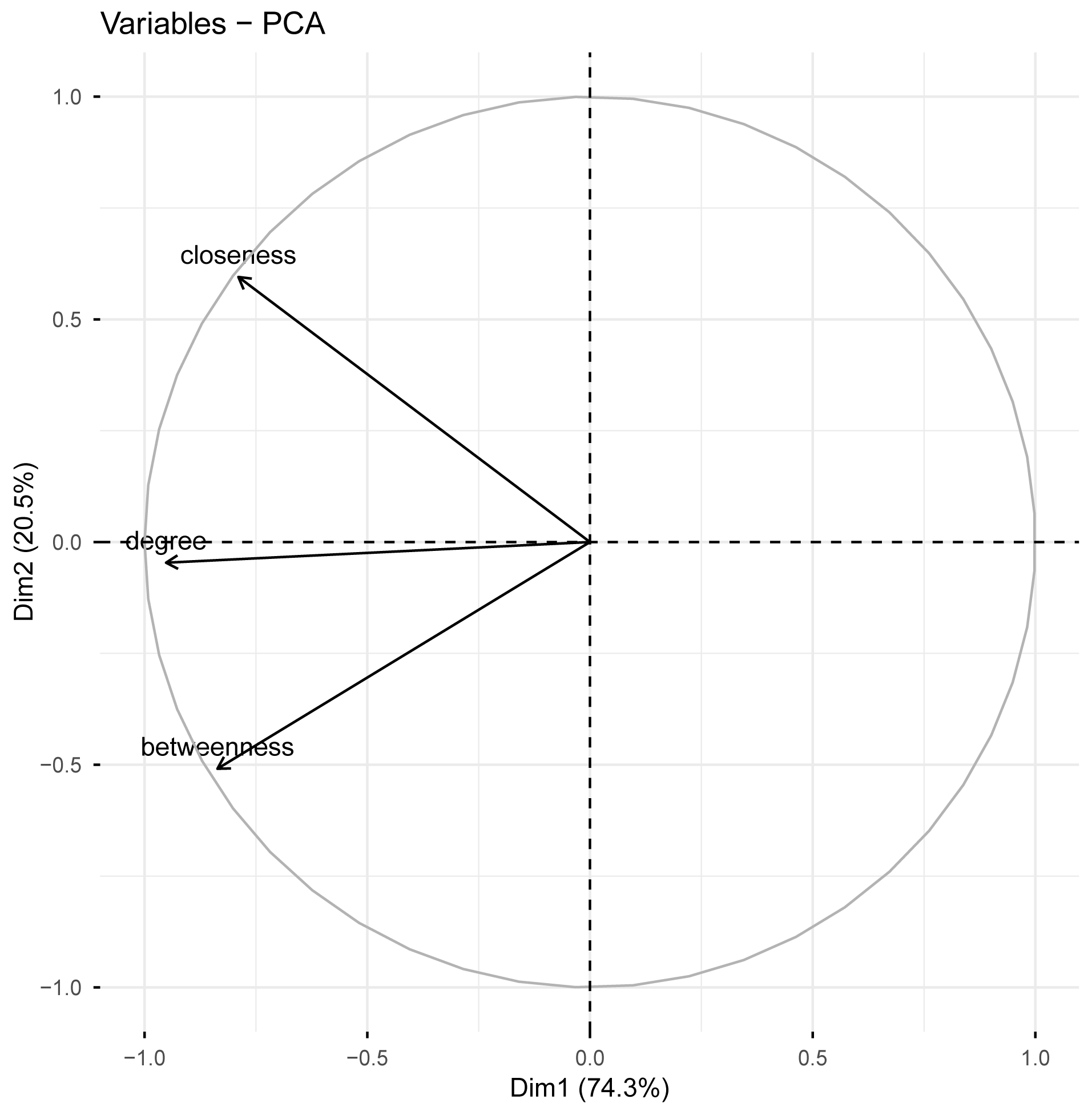


Figure S1. Principal Component Analysis (PCA) of bat species centrality metrics in bat-bat fly interaction networks. The biplot shows the loadings of degree, closeness, and betweenness centrality on the first two principal components. Dim1 explains 74.3% of the total variance and represents a general gradient of host importance, with all three centrality measures loading strongly and in the same direction. Dim2 explains 20.5% of the variance.


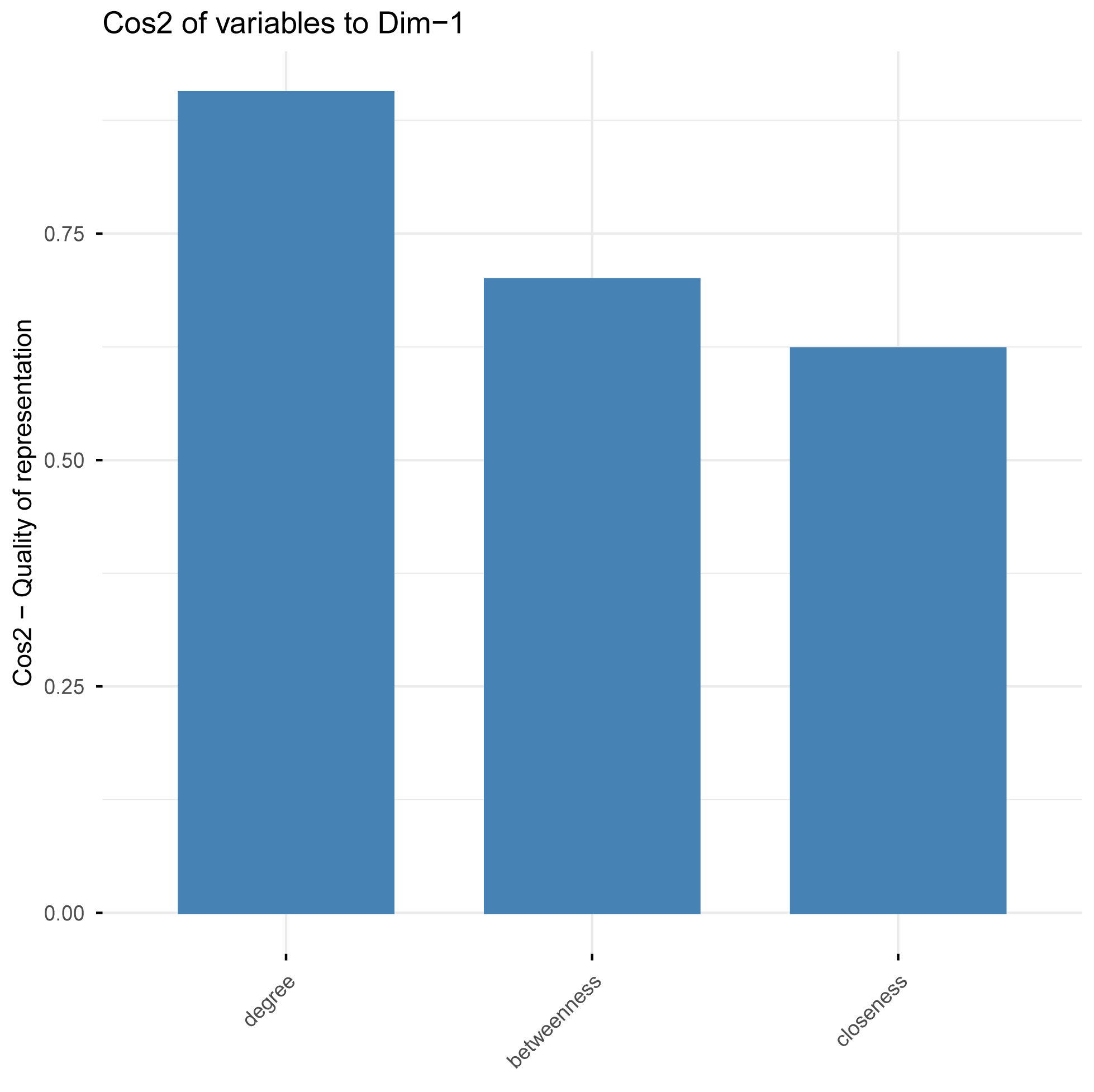


Figure S2. Quality of representation (squared cosine) of each centrality metric on the first principal component (PC1) of the PCA. Higher cos2 values indicate that a larger proportion of the variance of a given metric is captured by PC1. Degree centrality shows the highest contribution to PC1, followed by betweenness and closeness, confirming that PC1 primarily represents overall host importance in bat–bat fly interaction networks.


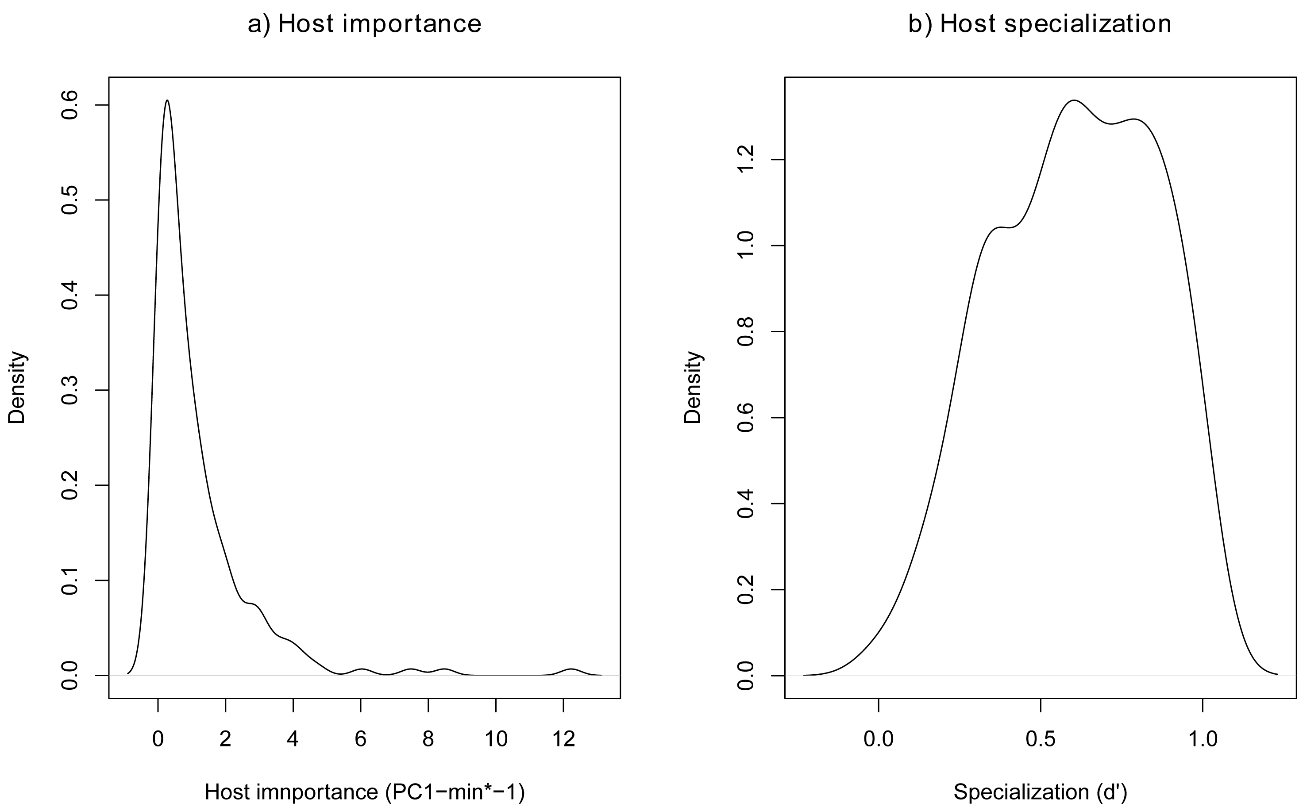


Figure S3. Density distributions of host importance (PC1-min*-1) and specialization (d) metrics used to guide the choice of error distributions in generalized additive mixed models testing the effects of host functional traits.
